# Mechanical Upcycling Immiscible Polyethylene Terephthalate-Polypropylene Blends with Carbon Fiber Reinforcement

**DOI:** 10.1021/acsapm.1c01850

**Published:** 2022-04-06

**Authors:** Andre N. Gaduan, Kanjanawadee Singkronart, Catriona Bell, Emma Tierney, Christoph Burgstaller, Koon-Yang Lee

**Affiliations:** † Department of Aeronautics, Imperial College London, South Kensington Campus, SW7 2AZ London, United Kingdom; ¥ School of Engineering, Brown University, Providence, Rhode Island 02912, United States; ‡ Transfercenter für Kunststofftechnik (TCKT) GmbH, Franz-Fritsch-Straße 11, 4600 Wels, Austria; § Institute for Molecular Science and Engineering (IMSE), Imperial College London, SW7 2AZ London, United Kingdom

**Keywords:** polymer blend, composite, lifecycle analysis, plastic waste, mechanical properties, fracture toughness

## Abstract

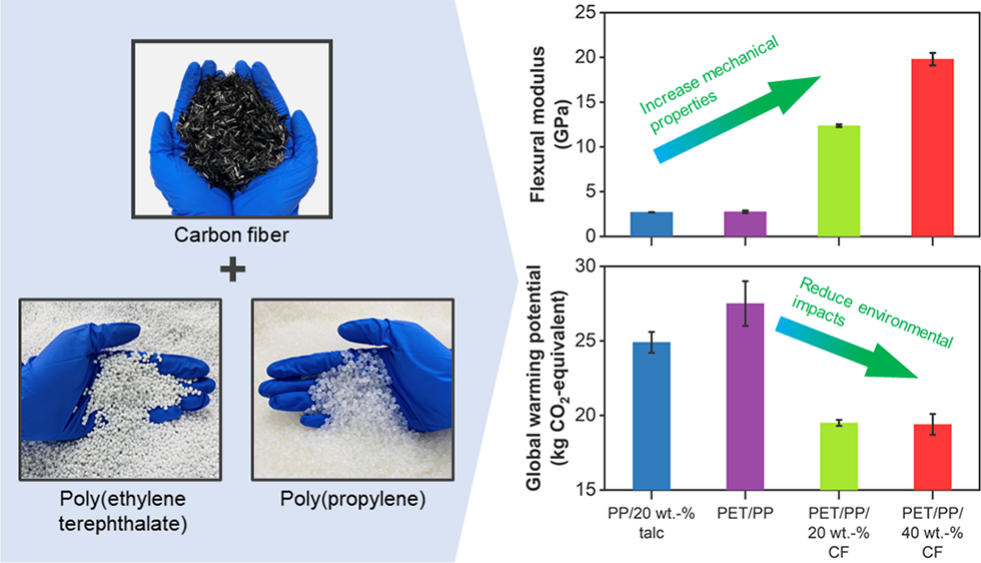

Ineffective sorting of post-consumer plastics remains one of the major obstacles in the recycling of plastics. Consequently, these highly heterogeneous, mixed post-consumer plastics will end up in landfill or have to be incinerated as repurposing them directly would lead to a polymer blend with inferior quality for many end-uses. In this work, we demonstrate the use of carbon fibers (CFs) to practically upgrade the mechanical properties of mixed plastics, adding value to them. This will create a stronger demand for mixed plastics to be used in various engineering applications. Using polyethylene terephthalate (PET) and polypropylene (PP) as the model immiscible polymer blend, we showed that the incorporation of CFs increased the tensile, flexural, and single-edge notched fracture toughness of the resulting CF-reinforced PET/PP composite blends. Despite the high environmental burden associated with the production of CFs, cradle-to-grave life-cycle analysis showed that CF-reinforced PET/PP composites have a lower environmental impact than the life-cycle scenarios of “doing nothing” and repurposing immiscible PET/PP blends as it is without CF reinforcement. This can be attributed to the weight saving achieved, a direct result of their higher mechanical performance. Our work opens up opportunities for the use of mixed plastics in various higher value applications such that they can be diverted away from landfill or incineration, in line with the concept of circular economy.

## Introduction

In July 2017, China announced to the World Trade Organization that it would forbid the importation of “foreign garbage”.^1^ Following this, China, who was the center of global recycling trade, implemented “Operation National Sword” on the 31st of December 2017, which abruptly ended the importation of waste materials, including a variety of post-consumer plastics. China now puts stringent monitoring and review in place such that only post-consumer plastics with a high degree of cleanliness will be accepted into the country for recycling. Consequently, the United Kingdom saw more than 90% decrease in the export of post-consumer plastics to China in the following year.^2^ Post-consumer plastics are now accumulating in local landfill, incinerated locally or shipped to developing countries that do not have the infrastructure nor the resources to properly dispose of them (i.e., the post-consumer plastics will still leak into the environment, just not in the United Kingdom).^3^ It is therefore crucial that we move away from a linear resource consumption model, e.g., “take-make-dispose”, and move toward a circular economy model, which focuses on turning post-consumer plastics into a resource.

According to a recent report produced under the U.K. Waste and Resource Action Programme (WRAP), ∼2.4 million tons of post-consumer packaging plastics are generated in the United Kingdom every year.^4^ However, only ∼1.1 million tons of these post-consumer plastics are collected and recycled (∼425,000 tons recycled in the United Kingdom and ∼650,000 tons exported to other countries for recycling).^5^ While more MRFs and PRFs can be built to increase the recycling rates in the United Kingdom, inefficient sorting remains a major barrier.^6−8^ As a result, the stream of highly heterogeneous mixed plastic waste ends up in landfill or is incinerated as it is no longer cost-effective to sort them.^7,9^ There is therefore a timely need to find value from this heterogeneous mixed plastic waste feedstock.

The easiest way to divert mixed plastic waste away from landfill or incineration is to repurpose it directly as it is. However, this will lead to a polymer blend with inferior quality for many end-uses as most polymers are incompatible and immiscible at the molecular level.^10^ The Flory–Huggins equation, which describes the Gibbs free energy of mixing (Δ*G*_mix_) of a binary blend of polymers, A and B, can be written as^11^
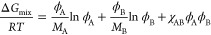
1where *R* is the universal gas constant, *T* is the temperature, and *M*_A_ and *M*_B_ are the degrees of polymerization of A and B, respectively. The terms ϕ_A_ and ϕ_B_ are the volume fractions of polymers A and B, respectively. χ_AB_ is the Flory–Huggins interaction parameter, which is a measure of the interaction between polymers A and B in the blend and can be estimated using the following equation:
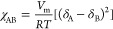
2where *V*_m_ is the mixing volume and δ_A_ and δ_B_ are the solubility parameter of A and B, respectively. A miscible blend will form if Δ*G*_mix_ ≤ 0. Since *M*_A_ and *M*_B_ are orders of magnitude larger than lnϕ_A_ and lnϕ_B_, Δ*G*_mix_ is predominantly governed by the magnitude of χ_AB_ (which is always ≥0). Only polymers with very similar δ values will yield a χ_AB_ ∼0 and hence, Δ*G*_mix_ ≤ 0. For most combination of polymers, however, Δδ is sufficiently large that an immiscible polymer blend is produced.^12^ This then leads to the formation of a heterogeneous morphology (e.g., sea-island or co-continuous structure), which acts as stress concentration points in the immiscible blend, leading to a deterioration in mechanical performance.^13^

The poor mechanical properties of an immiscibility polymer blend can often be mitigated with a compatibilizer. Compatibilizers work by lowering the interfacial tension between different polymers, stabilizing the dispersed phase against coalescence, and improving the adhesion between the different phases in the immiscible blend.^14^ For a comprehensive list of compatibilizers for different immiscible binary polymer blends, the readers are referred to the work of Maris et al.^9^ While compatibilizers do improve the mechanical properties of immiscible polymer blends, they are specific to the type of polymers in the blend. The effectiveness of compatibilizers is also sensitive to the composition of the different polymers in the blend.^15,16^ It is hard to predict the exact composition of polymers in any stream during the polymer recycling process, let alone a waste stream of mixed plastics, which is usually the remainder from inefficient plastic sorting.

To address this issue, some authors have explored a more pragmatic approach by using glass fibers (GFs)^17−22^ or natural fibers (NFs)^23−25^ to upgrade the mechanical performance of mixed plastics, thereby broadening their applications for various end-uses without the need to consider the exact composition of the different polymers in the batch of mixed plastics. The mechanical performance of the resulting glass or natural fiber-reinforced mixed plastics is dominated by the stronger reinforcing fiber instead of the inferior polymer matrix. Bajracharya et al.^21^ reported that a polymer blend of HDPE/LDPE/PP containing 30 wt % of GFs possessed a flexural strength and modulus of 48 MPa and 3.3 GPa, respectively, a significant improvement over the neat HDPE/LDPE/PP blend, which possessed a flexural strength and modulus of only 20 MPa and 0.7 GPa, respectively. Similarly, the incorporation of 60 wt % kenaf fiber increased the flexural modulus of the PP/PE blend by ∼600%, from 0.23 to 1.5 GPa.^24^ Improvements in tensile strength have also been reported when GFs are added into immiscible PET/HDPE^26^ and PET/PA-66^27^ blends. It should be noted however that the tensile properties of GF- and NF-reinforced polymer blends are still lower than those of conventional engineering polymers, such as ABS that has a tensile modulus and strength in the range of 2.1–2.8 GPa and 38–52 MPa,^28^ respectively, or bio-based polylactide that possesses a tensile modulus of ∼4 GPa and a tensile strength of ∼60 MPa.^29^

As carbon fibers (CFs) possess higher mechanical properties than GFs/NFs, the final CF-reinforced mixed plastics should possess better mechanical performance than those reinforced with GFs/NFs. This will create a stronger demand for mixed plastics to be used in various engineering applications, diverting them away from landfill or incineration. In this work, we demonstrate that CFs can be an effective reinforcement to upgrade the performance of, and thereby adding value to, mixed plastics. PET/PP blends are used as our model mixed plastics due to their immiscibility and their incompatibility in processing temperature, highlighting a possible “worst case scenario”. Furthermore, PET and PP are also the major components in the residue output streams of PRFs and MRFs.^30^ This present work focuses on the fabrication of CF-reinforced PET/PP composite blends and discusses the effect of CFs on the tensile, flexural, and fracture toughness responses of the resulting model PET/PP composite blends. A life-cycle assessment (LCA) is also conducted to quantify the environmental impact associated with the use of CFs to upgrade the properties of immiscible PET/PP blends.

## Experimental Section

### Materials

Polypropylene (PP) (HG313MO, Borealis AG, Austria) and polyethylene terephthalate (PET) (Polyclear, Indorama Ventures Polymers, Gersthofen, Germany) pellets were purchased from Borealis AG, Austria and Bigler AG, Switzerland, respectively. Chopped carbon fibers (Carbiso CT6, length = 6 mm) were purchased from ELG Carbon Fibre Ltd. (Coseley, UK). *n*-Dodecane (Merck, purity ≥99.0%), 1,4-dioxane (GPR RECTAPUR, purity ≥99.0%, stabilized with 25 ppm ionol), dimethyl sulfoxide (GPR RECTAPUR, purity ≥99.0%), formamide (TECHNICAL, purity ≥99.7%), and ethylene glycol (Reag. Ph. Eur., purity ≥99.0%) were purchased from VWR International Ltd. (Lutterworth, UK). All chemicals were used as received without further purification.

### Fabrication of Model PET/PP and Model CF-Reinforced PET/PP Blends

The various polymer and composite blends were processed using a co-rotating twin-screw extruder (Eurolab XL, Thermo Fisher Scientific, Karlsruhe, Germany) equipped with a 16 mm diameter screw. The extruder has a length-to-diameter ratio of 25, and a screw speed of 30 rpm was used during processing. Prior to materials fabrication, pellets of PP, PET, and chopped CFs were dry-mixed manually in batches of 500 g using a spatula at different mass ratios. For the fabrication of neat PET, PET/PP blends, and PET/PP/CF composite blends, the feeding zone of the extruder was set to be 280 °C while the temperatures of the four subsequent mixing zones were kept at 280, 275, 275, and 250 °C, respectively. A die temperature of 230 °C was used in the fabrication of these materials. Neat PP and PP/CF composite blends were fabricated at a lower temperature due to their lower melt viscosity. The temperature used in the feeding zone of the extruder was 180 °C, and the temperature in the four subsequent mixing zones was kept at 175 °C. A die temperature of 170 °C was used. All extrudates were then pelletized (Haake VariCut, Thermo Fisher Scientific, Karlsruhe, Germany) and injection-molded (Haake MiniJet Pro Piston Injection Molding System, Thermo Fisher Scientific, Karlsruhe, Germany) into dog bone (65 mm overall length, 10 mm gauge length, 3 mm thickness)- and rectangular (80 mm × 13 mm × 3 mm)-shaped test specimens. A mold temperature of 40 °C was used. For the injection molding of neat PET, PET/PP blends, and PET/PP/CF composite blends, the barrel temperature was set at 280 °C. For the injection molding of neat PP and PP/CF composite blends, the barrel temperature was set to be 190 °C. All samples were injection-molded at an injection pressure of 650 bar for 30 s followed by a post-pressure of 650 bar for a further 90 s.

### Materials Characterization

#### Scanning Electron Microscopy (SEM)

The morphology of the fabricated materials was investigated using a large chamber scanning electron microscope (Model S-3700 N, Hitachi, Tokyo, Japan). An accelerating voltage of 15 kV was used. Prior to SEM, the samples were mounted onto aluminum stubs using carbon tabs and Au coating (Automatic sputter coater, Agar Scientific, Stansted, UK) at a current of 40 mA for 20 s.

#### Contact Angle Measurements of PET and PP

The dispersive (γ_S_^d^) and polar (γ_S_^p^) surface energies of PET and PP were determined from the contact angle of various test liquids (see Table S1 of the Supporting Information) on film samples using sessile drop method (EasyDrop, Krüss GmbH, Hamburg, Germany). Prior to the measurement, PET and PP were hot-pressed (4122 CE, Carver Inc., Wabach, USA) at 240 and 190 °C, respectively, under a weight of 2 tons to produce polymer films of ∼0.3 mm in thickness. The polymer film was then affixed on a glass slide using double-sided tape. A liquid droplet of 10 μL was carefully deposited on the surface of the polymer film, and the sessile drop was analyzed using the ellipse fitting method (Krüss ADVANCE, version 1.9.0.8). An average of five measurements was taken for each type of test liquid. The γ_S_^d^ and γ_S_^p^ of PET and PP were calculated using the Owen–Wendt–Rabel–Kaelble (OWRK) approach.^31^

#### Mechanical Properties of the Fabricated Materials

Tensile, flexural (three-point bending), and single-edge notched fracture toughness properties of the samples were determined in accordance with ASTM D638-14, ASTM D790-17, and ASTM 5054-15, respectively. The tests were performed using a universal testing machine (Model 4502, Instron Corporation, High Wycombe, UK), and a total of four specimens were tested in each test. Prior to tensile testing, a dotted pattern was marked on the surface of the dog bone test specimen using a stamp (IMT-ACC001, iMetrum Ltd., Bristol, UK). The strain of the test specimen was then evaluated by monitoring the movement of these dots using a non-contact optical extensometer (iMetrum Ltd., Bristol, UK). A crosshead displacement speed of 1 mm min^–1^ (corresponding to a strain rate of 0.1% s^–1^) was used during tensile testing. Flexural test was conducted at a crosshead displacement speed of 10 mm min^–1^ and a span length of 50 mm (span-to-thickness ratio of 16). The deflection of the test specimen was evaluated by monitoring the movement of the loading pin using a non-contact optical extensometer (iMetrum Ltd., Bristol, UK). Single-edge notched fracture toughness of the fabricated materials was determined from single-edge notch beam (SENB) specimens. A notch with a depth of 6 mm was introduced at the halfway point lengthwise in the width direction of the test specimen using a band saw (Startrite 502S, A.L.T. Saws & Spares Ltd., Kent, UK). The notch was further sharpened by tapping a sharp scalpel at the tip of the notch. The initial crack length (*a*) to width (*w*) ratio, *x*, of the SENB test specimen was ∼0.54. The SENB test specimen was then loaded in three-point bending mode. A crosshead displacement speed and span of 1 mm min^–1^ and 50 mm were used, respectively. The initial stress intensity factor, *K*_IC_, of the SENB test specimen was calculated from
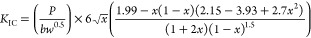
3where *P* is the load at crack initiation and *b* is the thickness of the test specimen.

#### Life-Cycle Assessment (LCA)

**Figure 1:**
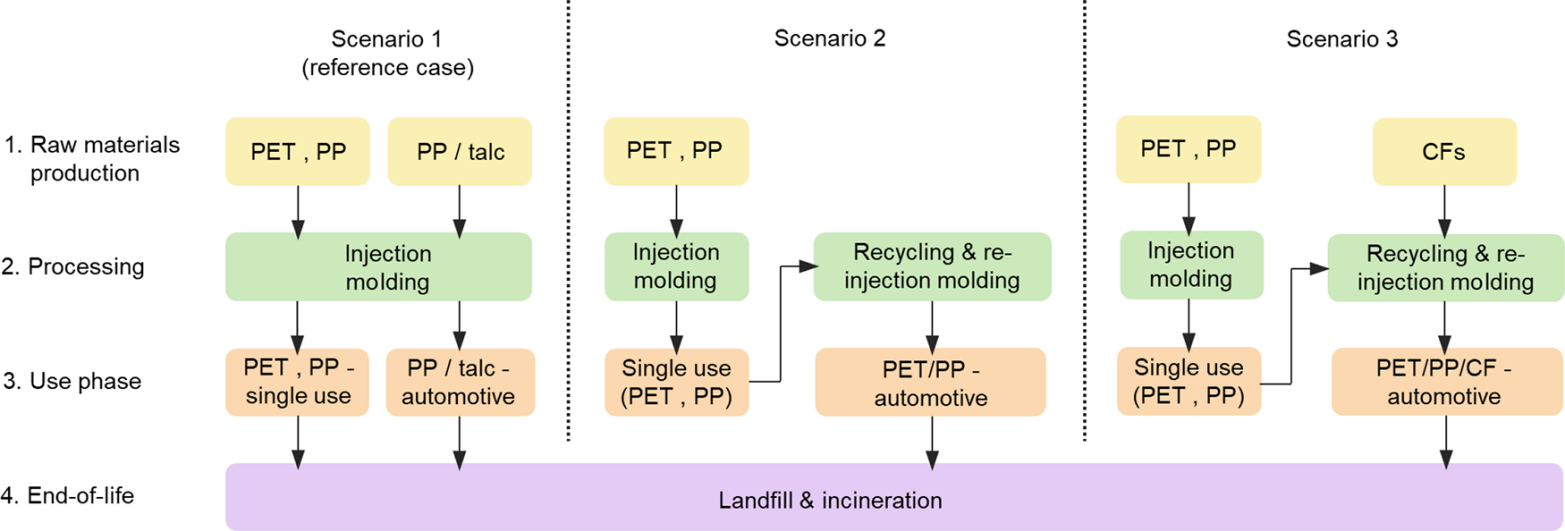
Schematic diagram showing the three scenarios modeled in our LCA model.

To ascertain whether CFs can be used to upgrade the properties of immiscible PET/PP blends and broaden their applications sustainably, LCA was conducted. The objective of this LCA is to quantify the environmental impact of PET/PP polymer blends and CF-reinforced PET/PP composite blends through a cradle-to-grave LCA, including the raw materials production, (re-)processing, use phase, and end-of-life. The functional unit (f.u.) of this LCA, which relates the environmental impact to the function of a product,^32^ is chosen as the equivalent mass of the fabricated material that is required to achieve the same level of flexural performance as 1 kg of PP filled with 20 wt % talc that is widely used in the automotive industry.^33−36^ A performance indicator based on the specific flexural modulus of the materials was used to calculate the mass of the functional unit (*m*_f. u._) required to achieve the same level of flexural performance as 20 wt % talc-filled PP. The term *m*_f. u._ can be calculated using
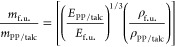
4where *E*_PP/talc_, ρ_PP/talc_, *E*_f. u._, and ρ_f. u._ are the flexural modulus and density of commercially available 20 wt % talc-filled PP (taken to be 2.7 GPa and 1.04 g cm^–3^)^37^ and the flexural modulus and density of the functional unit, respectively. The derivation of eq 4 can be found in the Supporting Information. Our LCA model considers three different life-cycle scenarios, and the system boundary is shown schematically in Figure 1.

Scenario 1 is our reference case, where virgin PET and PP are manufactured and disposed of at their end-of-life after single use, while 20 wt % talc-filled virgin PP is manufactured for automotive application and disposed of at its end-of-life. In scenario 2, virgin PET and PP are still manufactured and disposed of after single use but are diverted away from landfill/incineration. Instead, they are recovered as mixed plastic feedstock and reprocessed into PET/PP blends for use in automotive application before disposal. Consequently, the production of 20 wt % talc-filled virgin PP is avoided. In scenario 3, virgin CFs are added to the PET/PP blends to produce high-performance CF-reinforced PET/PP composites for automotive applications.

All data used in this study were taken from (i) the GaBi Professional database (version 9, Sphera Solutions GmbH, Leinfelden-Echterdingen, Germany), (ii) the literature, and (iii) our own estimations. A detailed inventory is included in the Supporting Information (Table S2). The electricity input of our LCA model is based on the European electricity mix. The energy required to extrude each functional unit (Δ*E*_total_) is estimated using^38^
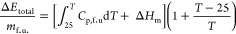
5where *C*_p, f. u_ is the specific heat capacity of the functional unit as a function of temperature, determined from differential scanning calorimetry (DSC), Δ*H*_m_ is the specific heat of fusion of the functional unit, and *T* is the processing temperature of the functional unit, which were 280 °C for neat PET, PET/PP, and PET/PP/CF blends and 190 °C for neat PP and PP/CF. The first term of eq 5 corresponds to the energy required to heat the functional unit from 25 °C to its processing temperature. The term  corresponds to the energy required to cool the functional unit from its processing temperature to 25 °C based on an ideal Carnot refrigeration cycle. The production of CFs is modeled based on the cradle-to-gate LCA of virgin CFs that covers the production of acrylonitrile, the conversion of acrylonitrile to polyacrylonitrile (PAN) precursor fibers, and the carbonization of PAN fibers to produce CFs.^39^ To evaluate the impact associated with the use phase, fuel consumption was allocated based on the weight of the functional unit. The car used in the LCA was modeled according to a Euro 1 passenger car with an engine size of 1.4 L, weighing 1500 kg and driven for 160,000 km. The fuel used during the use phase is based on the European gasoline mix. After the use phase, our LCA model assumes an end-of-life scenario consisting of 50% landfill and 50% incineration for energy recovery.^40^

Our LCA model uses the CML 2001 impact assessment method (January 2016 version) developed by the Centre for Environmental Science, Leiden University performed on the life-cycle engineering software, GaBi ts (version 9, Sphera Solutions GmbH, Leinfelden-Echterdingen, Germany). The chosen impact categories were global warming potential (GWP) and abiotic depletion potential of fossil fuel (ADPf). The following assumptions were made in our LCA model:

(i) Neat PP, neat PET, the various PET/PP blends, and CF-reinforced PET/PP composite blends were assumed to be equally durable in our LCA model.
(ii) The environmental impact associated with the transportation of materials were not considered.
(iii) The processing of 20 wt % talc-filled PP was assumed to be conducted at 190 °C.

## Results and Discussion

### Morphology of (CF-Reinforced) PET/PP Blends

**Figure 2:**
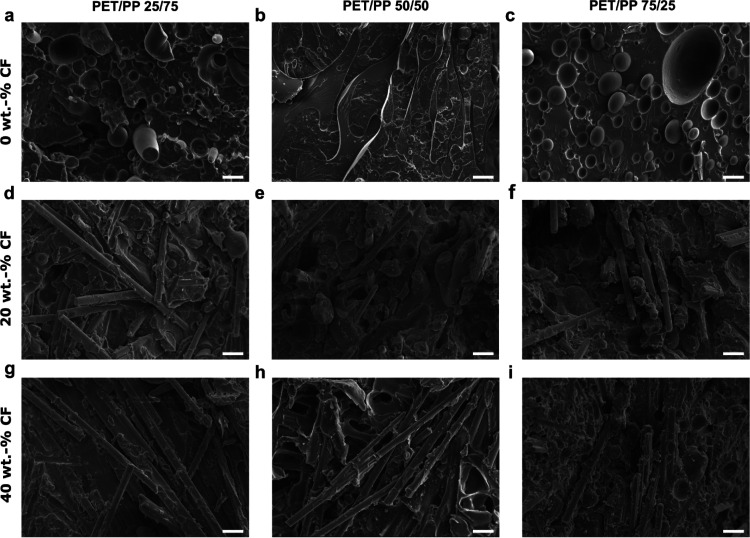
SEM images of the cryo-fractured surface of PET/PP blends and their respective CF-reinforced composite blends. (a-c) Neat PET/PP blends, (d-f) 20 wt.-% CF-reinforced PET/PP composite blends and (g-i) 40 wt.-% CF-reinforced PET/PP composite blends. Scale bar = 20 μm.

The internal morphology of the (CF-reinforced) PET/PP blends is shown in Figure 2. Neat PET/PP 25/75 blend (Figure 2a) exhibits a sea-island morphology, with the minor PET phase dispersed in the major PP phase as spherical domains. Increasing the PET content to a composition of PET/PP 50/50 leads to the formation of a co-continuous structure (Figure 2b). This is also indicative that a further increase in the PET content will lead to a phase inversion, which can be seen in Figure 2c for PET/PP 75/25. A sea-island morphology was again observed but with the minor PP phase dispersed in the major PET phase as spherical droplets. Phase separation in a polymer blend will occur if Δ*G*_mix_ > 0. The solubility parameters δ of PET and PP are 16.6 and 20.5 MPa^1/2^, respectively,^12^ and the Δδ value of a PET/PP blend is sufficiently large to produce an immiscible blend. Figure 2d–f show the internal morphology of the composite blends reinforced with 20 wt % CFs at various PET/PP compositions. The internal morphology of the composite blends reinforced with 40 wt % CFs at various PET/PP compositions is shown in Figure 2g–i. It can be seen from Figure 2d,g that, when the PET content is low (i.e., the PET/PP 25/75 blend), the incorporation of CFs leads to the disruption of the sea-island morphology that is evident in the neat PET/PP 25/75 blend. Such an effect was not observed when the PET content was increased to PET/PP 50/50 and PET/PP 75/25. The co-continuous and sea-island morphologies are still retained in these composite blends (Figure 2e,f for 20 wt % CF-reinforced and Figure 2h,i for 40 wt % CF-reinforced). To investigate the effect of CFs on the disruption of the sea-island morphology in the PET/PP 25/75 blend, wetting studies were conducted.

### Wetting of CFs by PP and PET

Table 1 summarizes the γ_s_^p^ and γ_s_^d^ of PET and PP calculated using the OWRK approach. The γ_s_ of PET and PP agrees well with the values reported in the literature.^41^ Both PET and PP possess similar γ_s_^d^. The higher polarity (defined as *X*^P^ = γ_s_^p^/γ_s_) of PET compared to PP is attributed to the presence of aromatic, ester, and hydroxyl groups in PET, while PP contains only non-polar methyl and methylene groups. Using the data in Table 1, we further estimated the thermodynamic work of adhesion (*W*_a_) between CFs and PET (*W*_a, CF/PET_) as well as CFs and PP (*W*_a, CF/PP_) using the equation
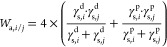
6where γ_s, *i*_^d^ and γ_s, *i*_^P^ as well as γ_s, *j*_^d^ and γ_s, *j*_^P^correspond to the dispersive and polar surface energy of components *i* and *j*, respectively. The surface energy of CFs was obtained from Bismarck et al.,^42^ which covers the range of surface energies for different CF surfaces. The estimated *W*_a, CF/PET_ and *W*_a, CF/PP_ are summarized in Table 1.

**Table 1:** Surface Energy of PET, PP, and CF Calculated Using the OWRK Method

sample	γ_s_ (mJ m^–2^)	γ_s_^p^ (mJ m^–2^)	γ_s_^d^ (mJ m^–2^)	*X*^P^	*W*_a, PP_ (mJ m^–2^)	*W*_a, PET_ (mJ m^–2^)
PET	35.0 ± 1.4	10.0 ± 1.8	25.0 ± 0.4	0.28 ± 0.04	57.6 ± 1.0	
PP	27.9 ± 0.3	2.1 ± 0.3	25.9 ± 0.2	0.07 ± 0.01		57.6 ± 1.0
CF^a^						
unsized	37.5 ± 2.3	10.0 ± 1.6	27.5 ± 1.7	0.27 ± 0.05	60.1 ± 0.9	72.3 ± 1.4
acidic	61.8 ± 4.5	39.8 ± 4.3	22.0 ± 1.5	0.64 ± 0.08	55.3 ± 1.2	78.6 ± 4.2
basic	47.1 ± 2.1	8.1 ± 1.6	39.0 ± 1.2	0.17 ± 0.01	68.7 ± 0.8	78.8 ± 0.9

a Data obtained from Bismarck et al.^42^

Higher *W*_a_ value corresponds to lower contact angle between the two phases and hence, better wettability. It can be seen from Table 1 that the *W*_a_ value between PET and PP is low. This is consistent with the incompatibility between the two polymers. The *W*_a, CF/PET_ value is higher than the *W*_a, CF/PP_ value, independent of the type of CF surface and higher than the *W*_a_ value between PET and PP. This implies that PET will preferentially wet out the CFs. As a result, the sea-island morphology that was previously evident in the neat PET/PP 25/75 blend was no longer observed when CFs were incorporated into the blend. The reappearance of the co-continuous and sea-island structures when the composition of PET was increased to PET/PP 50/50 and PET/PP 75/25 can be attributed to the low surface area of CFs (measured to be ∼0.24 m^2^ g^–1^),^43^ which led to insufficient CF surface area to be preferentially wetted out by PET.

### Tensile Properties of (CF-Reinforced) PET/PP Blends

**Figure 3:**
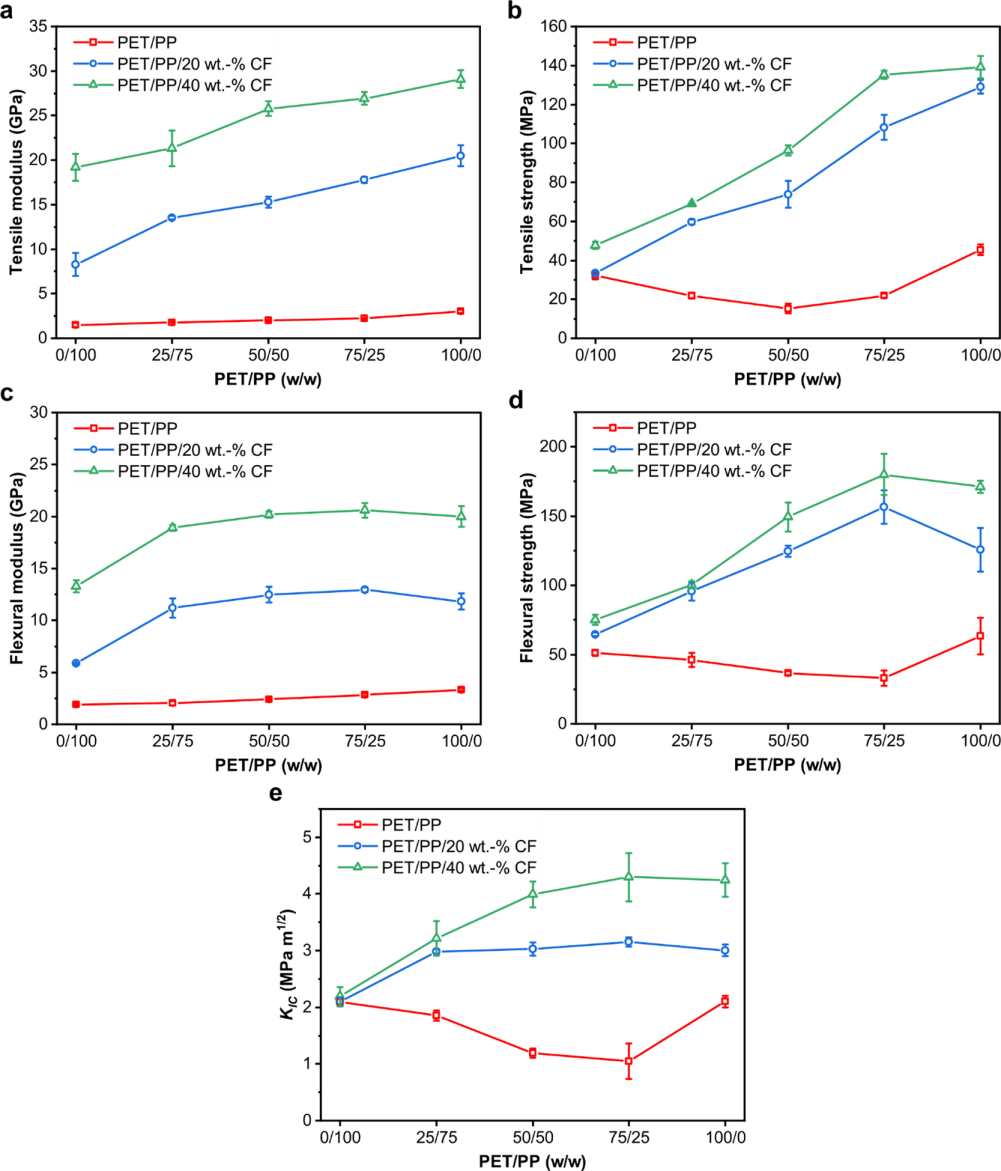
Mechanical properties of (CF-reinforced) PET/PP blends. (a) Tensile modulus, (b) tensile strength, (c) flexural modulus, (d) flexural strength, and (e) single-edge notched beam fracture toughness.

The tensile properties of the model (CF-reinforced) PET/PP blends are presented in Figure 3. Increasing the PET content in the PET/PP blend increases the tensile modulus from 1.5 GPa for neat PP to 3.0 GPa for neat PET (Figure 3a). The addition of CFs also has a positive effect on the tensile modulus of the CF-reinforced PET/PP composite blends. At 20 wt % CF loading, the tensile modulus of the fabricated materials increased linearly from 8.3 GPa for CF-reinforced PET/PP 0/100 to 20.5 GPa for CF-reinforced PET/PP 100/0. A further increase in the loading fraction of CFs to 40 wt % increases the tensile modulus from 19.2 GPa for CF-reinforced PET/PP 0/100 to 29.1 GPa for CF-reinforced PET/PP 100/0. The tensile strength of the fabricated materials (Figure 3b), on the other hand, showed a slightly different trend. Neat PET and PP possess a tensile strength of 45.5 and 32.1 MPa, respectively. However, the tensile strength of the PET/PP blends decreased to 21.8 MPa for PET/PP 25/75 and PET/PP 75/25. This is due to the presence of heterogeneous sea-island morphology and the poor adhesion between the different phases in the PET/PP blends, which acts as stress concentration points especially at the PET/PP interface, leading to early onset failure of the polymer blend.^13^ This effect was further exaggerated in PET/PP 50/50 where a co-continuous morphology was observed. The tensile strength of PET/PP50/50 decreased to only 15.2 MPa. When CFs were added into the PET/PP blends, the tensile strength of the CF-reinforced PET/PP blends increased linearly with increasing PET content. The highest tensile strength was attained when the matrix was PET/PP 100/0. This is postulated to be due to the better wettability between CFs and PET compared to CFs and PP (see Table 1). It is worth mentioning that the tensile properties of our CF-reinforced PET/PP composites are significantly higher than immiscible blends reinforced with NFs, which typically have a tensile modulus of 0.4–1.4 GPa and tensile strength of only 5–24 MPa.^44−46^ The tensile properties of GF-reinforced immiscible polymer blends are typically around 1.5–4.0 GPa in tensile modulus and 18–75 MPa in tensile strength,^21,26,47^ which are still lower than the CF-reinforced PET/PP composites prepared in this work.

### Flexural Properties of (CF-Reinforced) PET/PP Blends

Figure 3c,d summarize the flexural properties of (CF-reinforced) neat PET, PP, and PET/PP blends. Similar to the trend observed for the tensile modulus, the flexural modulus of the neat PET/PP blends increases with increasing PET content from 1.9 to 3.3 GPa. The addition of CFs also has a positive effect on the flexural modulus of all fabricated materials. The flexural modulus of neat PET and PP increased by up to 500 and 600%, respectively, when reinforced with 40 wt % CFs. When the PET content of the PET/PP blend increased to 25%, the flexural modulus increased by ∼91% for 20 wt % CF-reinforced and ∼42% for 40 wt % CF-reinforced PET/PP 25/75. Beyond this however, the flexural modulus of the CF-reinforced PET/PP composite blends plateaued at 12 and 20 GPa for 20 wt % and 40% CF reinforcement. As the tensile modulus of the CF-reinforced PET/PP composite blends was found to increase with increasing PET content (Figure 3b), the observed plateau flexural modulus can be attributed to a decrease in the compressive modulus when the PET content increases since the flexural properties of a material is a complex stress state that combines its tensile and compressive properties.^48^ The flexural strength of the CF-reinforced composite blends also increased when compared to neat PET, PP, and PET/PP blends (Figure 3d). An increment of 445% was observed when PET/PP 75/25 was reinforced with 40 wt % CF. Unlike the flexural modulus, however, the flexural strength of the CF-reinforced composite blends increased with increasing PET content and reached the highest flexural strength at 75 wt % PET content. This is attributed to the PP phase acting as a toughening agent and corroborates with the results that CF-reinforced PET possesses lower flexural strength than PET/PP 75/25. The flexural properties of the CF-reinforced PET/PP composites are also higher than those reported when NFs and GFs were used as reinforcement for immiscible polymer blends.^21,24,27,44,47^

### Fracture Toughness of (CF-Reinforced) PET/PP Blends

The *K*_IC_ values determined from SENB test specimens of neat PET, PP, and PET/PP blends as well as their respective CF-reinforced composites are shown in Figure 3e. Neat PET and PP possess a similar *K*_IC_ of ∼2 MPa m^0.5^. The immiscibility, heterogeneous morphology, and poor compatibility between PET and PP promoted the premature failure of the PET/PP blends, leading to the observed lower *K*_IC_ values than their pure polymer counterparts. The addition of fiber reinforcement often improved the fracture toughness of the resulting fiber-reinforced composite materials due to the introduction of additional energy absorbing mechanisms during fracture, including fiber/matrix debonding and fiber pull out.^49^ However, it can be seen from Figure 3e that the addition of CFs increased the fracture toughness of PET but not the fracture toughness of PP. The *K*_IC_ value of PET increased from 2.1 to 3.0 and 4.2 MPa m^0.5^ when 20 and 40 wt % CFs were added, respectively. The *K*_IC_ value of CF-reinforced PP remained constant at ∼2 MPa m^0.5^ with the addition of CFs. This can also be attributed to the better wettability between CFs and PET compared to CFs and PP (see Table 1). The better compatibility between PET and CFs also led to the increase in the *K*_IC_ values of the CF-reinforced PET/PP blends with increasing PET content.

### Life-Cycle Assessment of the Composite Panel Made from Model (CF-Reinforced) PET/PP Blends

We have demonstrated that CFs can be used to upgrade the mechanical properties of immiscible PET/PP blends, achieving a tensile modulus and strength of up to 29 GPa and 140 MPa, respectively, as well as a flexural modulus and strengh of 21 GPa and 180 MPa, respectively. However, the production of CFs is energy-intensive. It is estimated that the manufacturing of 1 ton of CFs produces 2400–3100 kg CO_2_-eq. For polyacrylonitrile (PAN)-based CFs, this high environmental burden stems from the production of PAN as well as the high energy consumption associated with stabilization and carbonization.^50^ On the contrary, the production of 1 ton glass or natural fibers produces ∼200 and ∼70 kg CO_2_-eq, respectively.^51^ Therefore, CFs may not be feasible from an environmental standpoint to upgrade the performance of immiscible polymer blends. Nevertheless, the higher mechanical properties of the fabricated CF-reinforced PET/PP composites than the benchmark 20 wt % talc-filled PP could lead to a significant weight saving (Table S3 in the Supporting Information) in the final composite part for use in automotive applications. The lighter the part, the lower the fuel consumption contributed by the part, and thus, less exhaust gas produced, which is beneficial to the environment overall.

**Figure 4:**
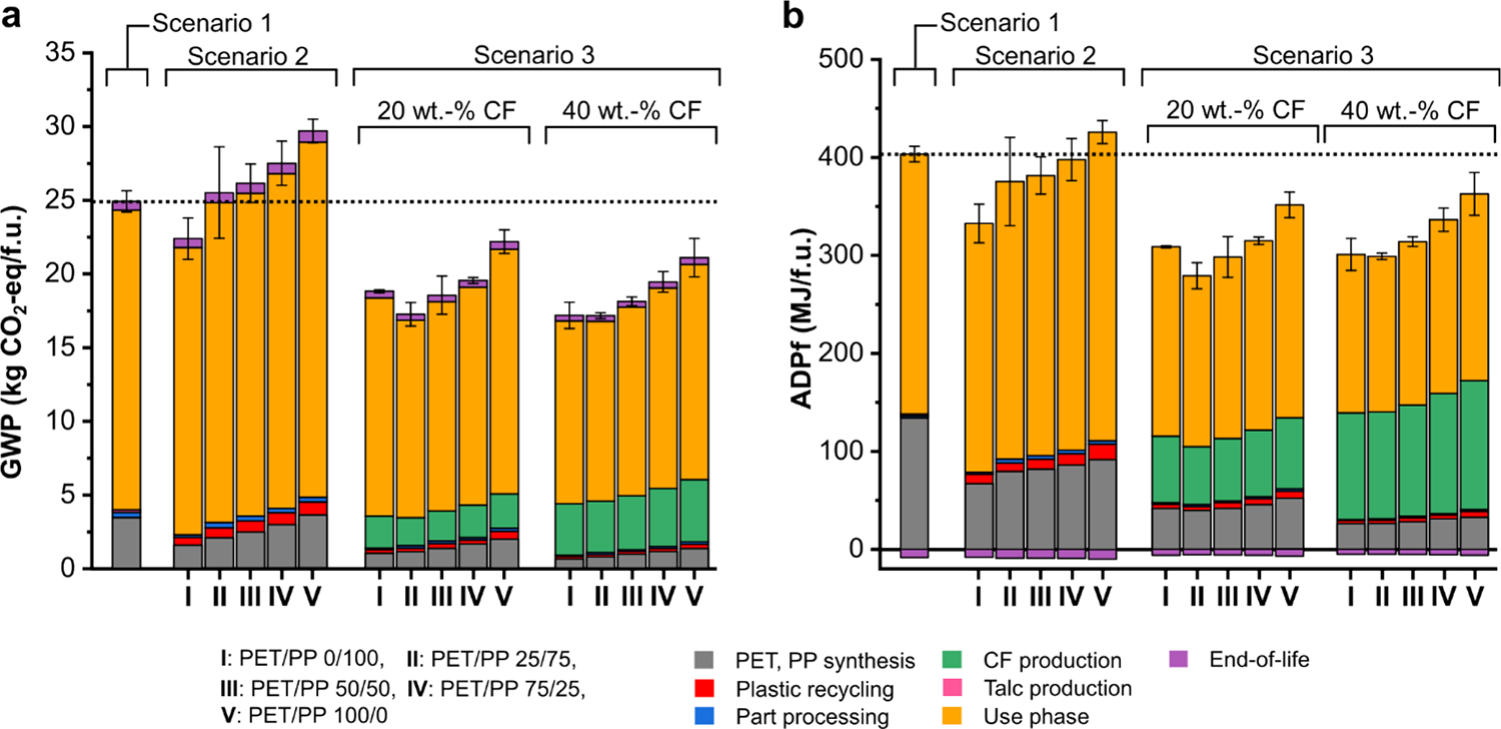
Cradle-to-grave (a) global warming potential (GWP) and (b) abiotic depletion potential (fossil fuel) (ADPf) of (CF-reinforced) PET/PP blends.

Figure 4 shows the cradle-to-grave GWP and ADPf associated with the raw materials production, processing, use phase, and the end-of-life of the (CF-reinforced) PET/PP blends in the various life-cycle scenarios described in the Life-Cycle Assessment (LCA) section. Our reference case (scenario 1 in Figure 4), where after their use phases, PET, PP, and 20 wt % talc-filled PP are landfilled and incinerated, contributes 25 kg CO_2_-eq./f.u. in GWP and 395 MJ/f.u. in ADPf. Here, the biggest contributor is the use phase of the 20 wt % talc-filled PP. As aforementioned, a heavier automotive part results in higher fuel consumption and consequently higher GWP. More energy is required to manufacture and mobilize a heavier part, which leads to a high ADPf. With the exception of PET/PP 0/100, the cascade recycling of single-use PET and PP for automotive applications leads to higher GWP (scenario 2 in Figure 4) than our reference case. This is due to the poor mechanical performance of the PET/PP blends, which requires a heavier part. The lower GWP of PET/PP 0/100 can be attributed to the lower density of PP and consequently higher weight saving.

When PET, PP, and PET/PP blends are reinforced with CFs (scenario 3 of Figure 4), both the GWP and ADPf are lower than our reference case (scenario 1) and the cascade recycling of PET and PP but without CF reinforcement (scenario 2). At 20 wt % CF loading, the GWP ranges between 19 and 22 kg CO_2_-eq./f.u and the ADPf ranges between 274 and 343 MJ/f.u. The lower GWP and ADPf values are a direct result of the significant improvement in the flexural modulus of the composites due to the introduction of CFs. This leads to a weight saving of 18–27% compared to 20 wt % talc-filled PP. As a result, both the environmental burden associated to raw materials, manufacturing, and fuel consumption during the use phase are reduced. It is worth mentioning that increasing the CF loading to 40 wt % did not result in any further reduction in both the GWP and ADPf values. While the 40 wt % CF-reinforced PET/PP composites possessed a higher flexural modulus, this increase is offset by the environmental burden of manufacturing CFs. Our LCA model showed that a CF loading of 20 wt % is the optimum to practically and sustainably upcycle immiscible PET/PP blend for automotive applications. At this CF loading, a good balance is achieved between the environmental burden of CF production, the mechanical performance, and hence, the reduced fuel consumption during the use phase of this composite part due to weight savings.

While the use of virgin CFs could increase the cost of the final CF-reinforced PET/PP composites, this could potentially be offset by using reclaimed CFs (rCFs), which currently face end-of-life management issues. Therefore, we performed a rough cost analysis for the manufacturing of the CF-reinforced composite blends based on rCFs as well as post-consumer PET and PP recyclates. The price of rCFs has been estimated to be £1.06/kg.^52^ The price post-consumer PET and PP recyclates are taken to be £1.41 and £0.30–£0.70/kg, respectively (personal communication). Using these values, the cost of 20 wt % rCF-reinforced post-consumer PET/PP composites is estimated to be £1.09–£1.51/f.u., depending on the PET-to-PP ratio. The cost is further reduced to £0.99–£1.26/f.u. for 40 wt % rCF-reinforced post-consumer PET/PP composites (see the Supporting Information for the cost breakdown of each composite formulation). This reduction is due to the lower mass of f.u. when 40 wt % fiber reinforcement is used. As a comparison, the cost of 20 wt % talc-reinforced PP composites is £1.72–£2.05/f.u,^28^ highlighting the cost savings achieved associated with the use of high-performance (reclaimed) carbon fiber as reinforcement to upcycle immiscible post-consumer mixed plastics.

## Conclusions

In this work, we showed that CFs can be used to upgrade the mechanical properties of immiscible PET/PP blends. Neat PET/PP blends possess an inferior tensile modulus and strength of only ∼1.8–2.2 GPa and 15–22 MPa, respectively, as well as a flexural modulus and strength of only ∼2.0–2.8 GPa and 33–46 MPa, respectively. The incorporation of 20 wt % CFs into PET/PP blends increased the tensile modulus and strength to 13.5–17.8 GPa and 60–108 MPa, respectively. The flexural modulus and strength of 20 wt % CF-reinforced PET/PP composites were found to be as high as 12 GPa and 157 MPa, respectively. A further increase in mechanical properties was also observed when the content of CFs was increased to 40 wt %. The cradle-to-grave LCA model estimated the GWP and ADPf of 20 wt % CF-reinforced PET/PP composites to be 19–22 kg CO_2_-eq./f.u. and 274–343 MJ/f.u., respectively. These values are lower than those of neat PET/PP (GWP = 22–30 kg CO_2_-eq./f.u., ADPf = 325–416 MJ/f.u.) and our benchmark material of 20 wt % talc-filled PP (GWP = 25 kg CO_2_-eq./f.u., ADPf = 395 MJ/f.u.). The lower environmental burden of CF-reinforced PET/PP composites can be attributed to the significant weight savings due to their higher mechanical performance.

## Supporting Information Available

Derivation of the flexural stiffness-driven design of a simply supported plate with concentrated force at the center, the dispersive (γ_LV_^d^) and polar (γ_LV_^p^) surface tension of test liquids used to determine the γ_s_ of PET and PP, life-cycle inventory of processes considered in the LCA, weight saving and mass of the functional unit of (CF-reinforced) PET/PP blends against 20 wt % talc-filled PP, and cost breakdown of the different composite formulations (PDF)
