# Supplementary material for: Mechanical Upcycling Immiscible Polyethylene Terephthalate-Polypropylene Blends with Carbon Fiber Reinforcement
Source: ACS Appl Polym Mater. 2022 Apr 6;4(5):3294–303. doi: 10.1021/acsapm.1c01850 (PMC9112279; doi:10.1021/acsapm.1c01850)
Supplement: Supplementary file 1 — ap1c01850_si_001.pdf [file ap1c01850_si_001.pdf]

SUPPORTING INFORMATION

Mechanical upcycling immiscible polyethylene terephthalate-polypropylene blends with carbon fibre reinforcement

Andre N. Gaduan[†], *Kanjanawadee Singkronart*[†], *Catriona Bell*[¥], *Emma Tierney*[†], *Christoph Burgstaller*[‡], *Koon-Yang Lee*[†§*]

[†]Department of Aeronautics, Imperial College London, South Kensington Campus, SW7 2AZ, London, United Kingdom

[¥]School of Engineering, Brown University, Providence, RI, 02912, USA

[‡]Transfercenter für Kunststofftechnik (TCKT) GmbH, Franz-Fritsch-Straße 11, 4600 Wels, Austria

[§]Institute for Molecular Science and Engineering (IMSE), Imperial College London, SW7 2AZ, London, United Kingdom

Number of pages = 8

Number of figures = 0

Number of tables = 4

Table of content

Derivation of flexural stiffness-driven design of simply supported rectangular plate with concentrated force at the centre Page S3

The dispersive ($\gamma_{\mathrm{LV}}^{\mathrm{d}}$) and polar ($\gamma_{\mathrm{LV}}^{\mathrm{p}}$) surface tension of test liquids used to determine the γ_{s} of PET and PP. Page S5

Life-cycle inventory of processes considered in the LCA Page S6

Weight saving and mass of functional unit of (CF-reinforced) PET/PP blends against 20 wt.-% talc-filled PP Page S7

Cost estimates of the various composite formulations Page S8

References Page S9

Derivation of flexural stiffness-driven design of simply supported rectangular plate with concentrated force at the centre

Consider the following loading case:

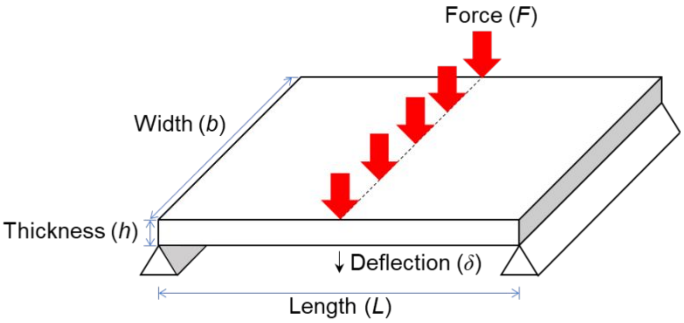

with the following design requirements:

Objective	To minimise the mass of the plate
Constraints	Length (L) and width (b) are fixed, while support bending load, F, without deflecting too much

The mass (m), stiffness (S) and deflection (δ) are:

$$m = hbL\rho \qquad \text{(S1)}$$

$$S = \frac{F}{\delta} = \frac{48EI}{L^3} \qquad \text{(S2)}$$

where E and I are the flexural modulus and second moment of area, respectively. The constraints require that:

$$\delta \leq \frac{FL^3}{48EI} \qquad \text{(S3)}$$

Since I of a rectangular plate is

$$I = \frac{bh^3}{12}, \qquad \text{(S4)}$$

and the stiffness of the plate can be written as:

$$S = \frac{48Ebh^3}{12L^3} = \frac{CEbh^3}{L^3} \tag{S5}$$

the stiffness (S), length (L) and width (b) are specified; thickness (h) is free. Therefore, the mass (m) of the plate can be minimized by reducing h.

$$h \geq \left(\frac{SL}{CEb}\right)^{1/3} \tag{S6}$$

Combining equations S1 and S6,

$$m \geq \left(\frac{SL^2b^{2/3}}{C}\right)\left(\frac{\rho}{E^{1/3}}\right) \tag{S7}$$

Given the constraints, the weight of the functional unit when compared to 1 kg of 20 wt.-% talc-filled PP is

$$\frac{m_{\text{f.u.}}}{m_{\text{PP/talc}}} = \left[\left(\frac{E_{\text{PP/talc}}}{E_{\text{f.u.}}}\right)^{1/3}\left(\frac{\rho_{\text{f.u.}}}{\rho_{\text{PP/talc}}}\right)\right] \tag{S8}$$

Table S1: The dispersive (γ_{LV}^{d}) and polar (γ_{LV}^{p}) surface tension of test liquids used to determine the γ_s of PET and PP. Data taken from Krüss ADVANCE software database (version 1.9.0.8).

Test liquids	γ_{LV}^{d} (mJ m^{-2})	γ_{LV}^{p} (mJ m^{-2})
n-dodecane	0	25.4
1,4-dioxane	0	33.0
Dimethyl sulfoxide	8.6	34.9
Formamide	19.0	39.0
Ethylene glycol / water (20:80 w/w)	43.4	20.9
Water	51.0	21.8

Table S2: Life-cycle inventory of processes considered in the LCA

**Process name**	**GWP (kg CO_2-eq.)**	**ADPf (MJ)**	**Reference/database**
Production of 1 kg PP	1.63	71.0	GaBi Professional
Production of 1 kg PET	2.88	75.4	GaBi Professional
Manufacturing of 1 kg talc	0.29	3.49	GaBi Professional
Manufacturing of 1 kg CF	14.60	454	[1]
Collection, sorting, and recycling of 1 kg post-consumer PET	0.91	14.9	[2]
Collection, sorting, and recycling of 1 kg post-consumer PP	0.53	8.88	[2]
Use phase (automotive) – 160 000 km	20.30	264.7	GaBi Professional
Incineration of 1 kg plastic waste	1.19	-17.7	GaBi Professional
Landfilling of 1 kg plastic waste	0.07	1.0	GaBi Professional
**Background process**			
Electricity grid mix (EU)	0.392	4.38	GaBi Professional
Gasoline mix (EU)	0.661	50.1	GaBi Professional

Table S3: Weight saving and mass of functional unit of (CF-reinforced) PET/PP blends against 20 wt.-% talc-filled PP

**Blend ratio, PET/PP (w/w)**	**Weight saving of panel based on *E*-driven design (%)**	**Mass of f.u. based on *E*-driven design (kg)**
**0 wt.% CF**		
100/0	+ 19.1	1.19
75/25	+ 12.4	1.12
50/50	+ 8.0	1.08
25/75	+ 6.8	1.07
0/100	− 3.8	0.96
**20 wt.-% CF**		
100/0	− 17.7	0.82
75/25	− 27.0	0.73
50/50	− 30.2	0.70
25/75	− 33.6	0.66
0/100	− 27.2	0.73
**40 wt.-% CF**		
100/0	− 28.5	0.72
75/25	− 33.1	0.67
50/50	− 37.1	0.63
25/75	− 39.7	0.60
0/100	− 39.0	0.61

Table S4: Cost estimates of the various composite formulations.

The analysis was conducted based on the use of reclaimed carbon fibres (rCFs) and post-consumer PET/PP recyclates. The price of rCFs was taken as £1.06/kg based on estimation by Meng et al.[3] The price of post-consumer PET and PP was taken as £1.41/kg and £0.30-£0.70/kg, respectively, based on a personal communication with a plastics recycler. The energy cost (£0.12/kWh) was taken from Eurostat.[4]

**Composite formulation**	**Total cost (£/f.u.)**
75/25 PET/PP + 20 wt.-% CF	1.43 – 1.51
50/50 PET/PP + 20 wt.-% CF	1.26 – 1.38
25/75 PET/PP + 20 wt.-% CF	1.09 – 1.25
75/25 PET/PP + 40 wt.-% CF	1.22 – 1.26
50/50 PET/PP + 40 wt.-% CF	1.09 – 1.16
25/75 PET/PP + 40 wt.-% CF	0.99 – 1.10

References

(1) Meng, F.; McKechnie, J.; Turner, T. A.; Pickering, S. J. Energy and Environmental Assessment and Reuse of Fluidised Bed Recycled Carbon Fibres. *Compos. Part Appl. Sci. Manuf.* **2017**, *100*, 206–214. https://doi.org/10.1016/j.compositesa.2017.05.008.

(2) *Life Cycle Impacts for Postconsumer Recycled Resins: PET, HDPE, and PP*. https://plasticsrecycling.org/images/library/2018-APR-LCI-report.pdf (accessed 2021 -04 -21).

(3) Meng, F.; McKechnie, J.; Pickering, S. J. An Assessment of Financial Viability of Recycled Carbon Fibre in Automotive Applications. *Compos. Part Appl. Sci. Manuf.* **2018**, *109*, 207–220. https://doi.org/10.1016/j.compositesa.2018.03.011.

(4) *Electricity price statistics*. https://ec.europa.eu/eurostat/statistics-explained/index.php?title=Electricity_price_statistics#Electricity_prices_for_non-household_consumers (accessed 2022 -02 -16).
